# Book Notices

**Published:** 1858-03

**Authors:** 


### BOOK NOTICES.

The Principles and Practice of Obstetrics, including the Treatment of Chronic Inflammation of the Uterus, considered as a Frequent Cause of Abortion. By Henry Miller, M. D., Prof. of Obstetric Medicine in the Medical Department of the University of Louisville. Blanchard & Lea, Philadelphia, 1858.

We like this work of Prof. Miller's, for the independent and manly originality of matter and expression. It contains more than an ordinary amount of originality for books of the present day. We like it all the better, because it is Western. It is a direct assurance to the western student, that the hot-beds of vice in Europe are not the only localities in which uterine infirmities may be studied with profit to the profession; and what is of equal importance, that the treatment for one latitude is applicable to that of another in this respect. Dr. Miller is the acknowledged and decided champion of the Bennet pathology of the uterus and its appendages on this side of the Atlantic. His views command the respect of all who have read them; they bear the impress of independent research, and should be read by every western physician with pride. While it could not be expected that we should subscribe to everything we find in Prof. Miller's book, we most heartily recommend it as one of the most reliable for consultation on the subjects of which it treats with which we are acquainted.

We should be glad to particularize some of the best points of our author, if space would permit, but it would require too much room, and it is not an easy matter to decide between selections where there are so many that would be very interesting.

A Practical Treatise on Diseases of Children. By J. Forsyth Meigs, M.D. Third Edition. Lindsay & Blackiston: Philadelphia, 1858.

The appearance of the third edition of Dr. Meigs' work in ten years is a pretty good evidence of the approval it has received. There is sufficient substance in the work to live still many years, under the judicious guidance of its indefatigable author. Many more sickly offspring have grown into such strength and lively robustness under his care, as to give promise of unusual longevity. Although not intended to be a complete work on diseases of children, it *has its own excellences*. The chapter of entero-colites is very interesting, and we think equal, if not superior to, anything on the same subject within our knowledge. His success as an author is placed beyond question, by the appearance of this beautiful and solid edition of his work on children.

THE PHYSICIAN'S HANDBOOK OF PRACTICE AND MEMORANDA FOR 1858; containing a Classified List of Diseases, with their Symptoms, Complications, etc.; an Alphabetical List of Remedial Agents, with their Properties, Preparations and Doses; a Classified List of Poisons, with their Symptoms and Antidotes; Examples of Extemporaneous Prescriptions, and Abbreviations of the Terms Used in Prescribing, with their Translations into English; to which is added a Record for Daily Practice. Prepared for the Names of Thirty or Sixty Patients, and other Memoranda. Second Edition. By WM. ELMER, M.D., and LEVI REUBEN, M.D. New York: Stringer & Townsend, No. 222 Broadway. 1858.

Such is the title-page in full of a neat little book which we have received from the authors. It is proposed another year to alter the arrangement in several particulars, one of which is to furnish practitioners with a key, by the aid of which they may find anything they wish in the various periodicals of the day. There are certainly several important items of interest to the busy practitioner in this little volume. The objection we have to it is, that it contains too much for a pocketbook, and too little for a combined case and day book. The man who is engaged in active locomotion likes to rid himself of every weight and hindrance, and even Lindsay & Blackiston's visiting list is getting rather cumbersome. We believe that the authors of the handbook might make a combined day and case book that would give room for legible record of the pecuniary and professional interest we have in our patients, that would better supply the wants of the profession than can be done by their present production. Anything we carry in our pocket we want of small space and ponderosity. But if a book is to lie in our desk, for daily record and reference, we have less concern for its size and weight than for its correctness, fulness and completeness. While we feel constrained to make the above observations, partly in reference to the handbook, we are free to recommend it as an excellent and comprehensive little remembrancer and daily record, to such as are willing to carry a very light package about them constantly.

We have only space to mention the reception of an "Introductory Address delivered before the Class of the Medical Department of the Iowa University, at the opening of the Course of Lectures for 1857–8, by A. T. Hudson, M.D., Prof. of Theory and Practice of Medicine."

"The Fifth Annual Report of the Surgeons of the New York Ophthalmic Hospital, with an Address by Mark Stephenson, M. D. (one of its Surgeons) to the Graduating Class." This institution is doing good service toward exterminating quackery, by educating regular physicians more thoroughly in this much-neglected specialty.

"First Annual Report of the Trustees and Superintendent of the Ohio Asylum for the Education of Idiotic and Imbecile Youth, to the Fifty-Third General Assembly, for the year 1857." This institution is situated at Columbus, Ohio, and is under the superintendence of R. J. Patterson, M.D., formerly of the Indiana Insane Asylum. We know of no man who is more capable of the duties of that difficult position than Dr. R. J. Patterson.

"Pliny Earle's very clear, philosophical and highly-erudite Medical Opinion in the Parish Will Case," is received.

We have also received the "Parish Will Case before the Surrogate of the City of New York," with the "Medical Opinions upon the Mental Capacity of Mr. Parish, by John Watson, M.D., D. T. Brown, M.D., M. H. Rauney, M.D., Pliny Earle, M.D., Luther V. Bell, M. D., L.L.D., J. Ray, M.D., Sir Henry Holland, Bart., M.D., F.R.S." A portly volume of 573 pages. Although so formidable in appearance as to discourage the timid from undertaking its perusal, the time thus spent would be well repaid by the valuable information contained in it.

